# A meta-analysis of the association between IL28B polymorphisms and infection susceptibility of hepatitis B virus in Asian population

**DOI:** 10.1186/s12876-015-0286-2

**Published:** 2015-05-12

**Authors:** Jing Chen, Wei Wang, Xiaoguang Li, Jie Xu

**Affiliations:** Department of Infectious Disease, Peking University Third Hospital, 100191 Beijing, China

**Keywords:** Meta-analysis, IL28B, rs12979680, rs8099917, HBV, Infection susceptibility

## Abstract

**Background:**

Several association studies with small sample sizes of two SNPs in IL28BB (rs12979860 and rs8099917) showed inconsistent results, so in this study, we aim to evaluate the association between the two SNPs and infection susceptibility of Hepatitis B Virus (HBV) in Asian population, especially in Chinese population by meta-analysis.

**Methods:**

Search the relevant published papers and perform meta-analysis respectively on IL28B (rs12979860 and rs8099917) in Asian population and Chinese population under an additive genetic model by STATA11.0.

**Results:**

The pooled odds ratios (OR) of rs12979860 are 0.79 (95% CI, 0.53-1.18; P = 0.25, I^2^ = 63.2%) and 1.62 (95% CI, 1.04-2.51; P = 0.033, I^2^ = 54.3%) respectively in Asian population and Chinese population analysis. The pooled OR of rs8099917 are 1.05 (95% CI, 0.93-1.19; P = 0.44, I^2^ = 43.3%) and 0.97 (95% CI, 0.84-1.23; P = 0.726, I^2^ = 15.6%) respectively in Asian population and Chinese population analysis.

**Conclusion:**

Our study demonstrated that T allele of rs12979680 can increase the risk of HBV infection in Chinese population but not Asian population under an additive genetic model. There is no association between rs8099917 and HBV infection in Chinese population and Asian population.

## Background

Hepatitis B virus (HBV) is a worldwide health problem, and it is estimated that 2 billion individuals have been infected with HBV and 350 million are the chronic carriers. In the data of World Health Organization (WHO) in 1997, the rate of chronic HBsAg carriers in Asian is higher compared with other continents, HBsAg seroprevalence in the north and central Asian countries ranged between 10% and 12% and in Southeast Asia it ranged between 1% and 10% with a total number of 130 million. But in the United States and northern European countries, it is under 0.5% [1]. In the Chinese epidemiological investigation of HBV in 2006, the rate of chronic HBsAg carriers was 7.18%, and it is about 93 million patients with HBV chronic infection [2]. With the development of human genome research, there is strong evidence in HBV infection that host genetic factors play a major role in determining the outcome of infection. The incidence of hepatitis B in Asian is high, so there may be genetic susceptibility in Asian people. The researchers have done much study on this issue in recent years, and Interleukin 28B (IL28B) has been a hot research topic on hepatitis virus, especially hepatic C virus. IL28B plays an important role in the outcome of hepatic C virus by genome-wide association studies (GWAS) [3] and it could inhibit hepatitis B and C virus replication [4]. HBV and HCV share some similarities in pathogenesis, natural history and acquired immune responses, but the relationship between ILB28 and HBV is still unclear. Rs12979860 and rs8099917 were widely studied in the previous research, so in this study, we performed a meta-analysis on single nucleotide polymorphisms (SNPs) of IL28B (rs12979860 and rs8099917) to evaluate the relationships between the genetic loci and HBV in Asian population, especially in Chinese population.

## Methods

### Identification of eligible studies

**Figure 1:**
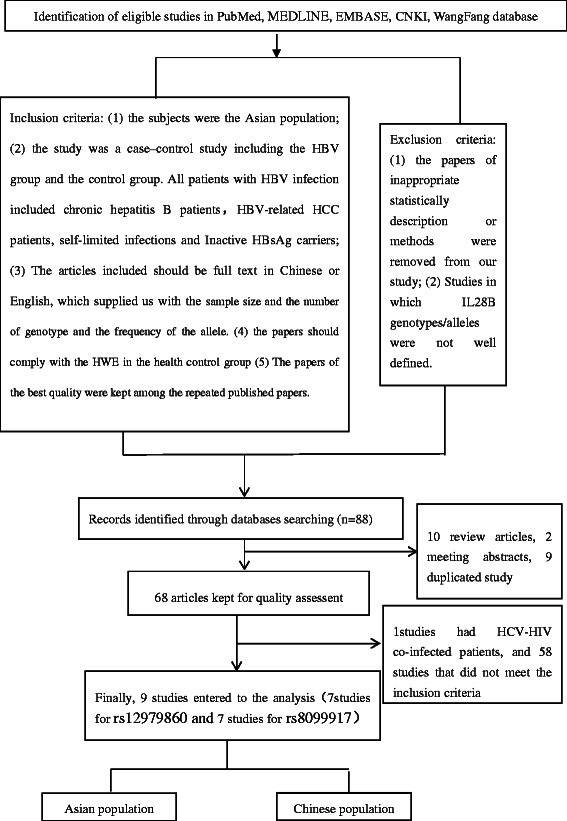
The flowchart of the meta-analysis.

We searched the databases including PubMed, MEDLINE, EMBASE, China National Knowledge infrastructure (CNKI) and WanFang database for case–control studies on IL28B published in Asian population up to March, 2015. The search terms included IL28B and Asian and HBV. Then we selected the papers included IL28B-rs12979860 and rs8099917 (Figure 1, Tables 1 and 2).

**Table 1:** **Characteristics of the papers included in the meta-analysis and allele frequency of IL28B rs12979860 (C-T)**

SNP	Area	Author	Year	allele frequency of HBV infection (N = 1,052)		allele frequency of control group (N = 2,823)	
				C	T	C	T
rs12979860	China	Shi et al. [16]	2011	153	11	34	0
	China	Li et al. [10]	2011	760	104	382	24
	China	Chen et al. [17]	2012	2238	172	455	33
	China	Ren et al. [12]	2012	236	46	45	2
	China	Lv et al. [18]	2012	488	36	525	29
	Korea	Kim et al. [15]	2013	707	41	185	15
	Thai	KimKong et al. [19]	2015	398	24	304	24
Sum				4,980	434	1,930	127

**Table 2:** **Characteristics of the papers included in the meta-analysis and allele frequency of IL28B rs rs8099917 (T-G)**

SNP	Area	Author	Year	allele frequency of HBV infection (N = 3,613)		allele frequency of control group (N = 1,409)	
				T	G	T	G
rs8099917	China	Li et al. [10]	2011	764	48	385	21
	China	Chen et al. [17]	2012	2276	134	461	27
	China	Ren et al. [12]	2012	248	34	45	2
	Korea	Kim et al. [15]	2013	852	38	196	16
	Thai	Kimkong et al. [19]	2015	402	20	303	25
	China	Jiao et al. [11]	2011	643	41	276	12
	China	Ma et al. [20]	2012	1494	90	947	67
Sum				6,679	405	2,989	170

### The criteria of articles included and excluded in the meta-analysis

The articles included in the study should meet the following criteria: (1) the subjects were the Asian population; (2) the study was a case–control study including the HBV group and the control group. All patients with HBV infection were selected for the study including chronic hepatitis B patients, HBV-related HCC patients and inactive HBsAg carriers; (3) The articles included should be full text in Chinese or English, which supplied us with the sample size and the number of genotype and the frequency of the allele. (4) The papers should comply with the Hardy-Weinberg equilibrium (HWE) in health control group (5) Paper of the best quality was remained among the repeated published papers. (6) The papers should have been performed with the ethical approval and patient consent.

The criteria of articles excluded from the study were: (1) The papers of inappropriate statistically description or methods were removed; (2) Studies in which IL28B genotypes/alleles were not well defined (Figure 1).

### Data extraction and analysis

We extracted data from the papers included (ethnicity, genotypic distribution and allelic frequency) and performed meta-analysis by STATA, version11.0 under an additive genetic model. (Tables 2 and 3) The heterogeneity was assessed by Cochran Q test (P <0.1 was considered to be significant). The heterogeneity was classed by I^2^ statistical test (I^2^ < 25%: low heterogeneity; I^2^ = 25–50%: moderate heterogeneity; I^2^ = 50–75%: high heterogeneity; I^2^ > 75%: extreme heterogeneity value) [5]. If P > 0.1 in Cochran Q test, the pooled ORs and 95% CIs were calculated in fixed effect model; otherwise in random effect model. Publication bias was evaluated by Begg’s test (P < 0.05 was considered to be significant).

**Table 3:** **The result of meta-analysis of rs12979680 and rs8099917**

SNPs	Ethnicity	Model	Pooled OR (95%CI)	P	Heterogeneity		Publication bias	
					I^2^	P	ɀ	P
rs12979680	Asian	Random	0.79 (0.53-1.18)	0.25	63.2%	<0. 1	0.30	0.76
rs12979680	Chinese	Random	1.62 (1.04-2.51)	0.033	54.3%	<0. 1	0.73	0.46
rs8099917	Asian	Fixed	1.05 (0.93-1.19)	0.44	43.3%	>0.1	0.9	0.37
rs8099917	Chinese	Fixed	0.90 (0.84-1.81)	0.726	15.6%	>0.1	1.71	0.09

## Results

### Results of meta-analysis of rs12979860 in Asian population

**Figure 2:**
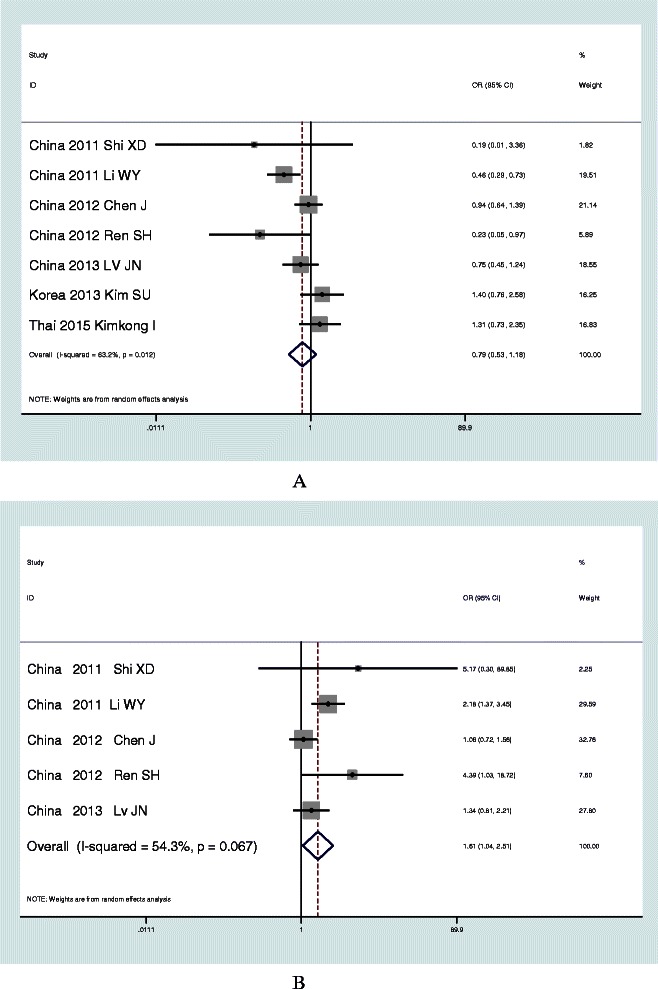
Meta-analysis results of rs12979680(C-T) **Panel A**. Meta-analysis results of rs12979680(C-T) in Asian population. **Panel B**. Meta-analysis results of rs12979680(C-T) in Chinese population.

In the meta-analysis of rs12979860 in Asian population, the pooled sample included 2,823 subjects with HBV infection and 1,052 control subjects from 7 studies in Asian population. There was high heterogeneity among studies by Cochran Q test (P < 0.1, I^2^ = 63.2%) and a random effect model was performed under an additive model. The pooled OR of risk allele T was 0.79 (95% CI, 0.53-1.18; P = 0.25). No publication bias was found in the meta-analysis (corrected ɀ = 0.30 and corrected p = 0.76) (Figure 2A, Table 3).

### Results of meta-analysis of rs12979860 in Chinese population

In the meta-analysis of rs12979860 in Chinese population, the pooled sample included 2,238 subjects with HBV infection and 788 control subjects from 5 studies in Chinese population. There was no heterogeneity among studies by Cochran Q test (P < 0.1, I^2^ = 54.3%) and a random effect model was performed under an additive model. The pooled OR of risk allele T was 1.62 (95% CI, 1.04-2.51; P = 0.033). No publication bias was found in the meta-analysis (corrected ɀ = 0.73 and corrected p = 0.46) (Figure 2B, Table 3).

### Results of meta-analysis of rs8099917 in Asian population

**Figure 3:**
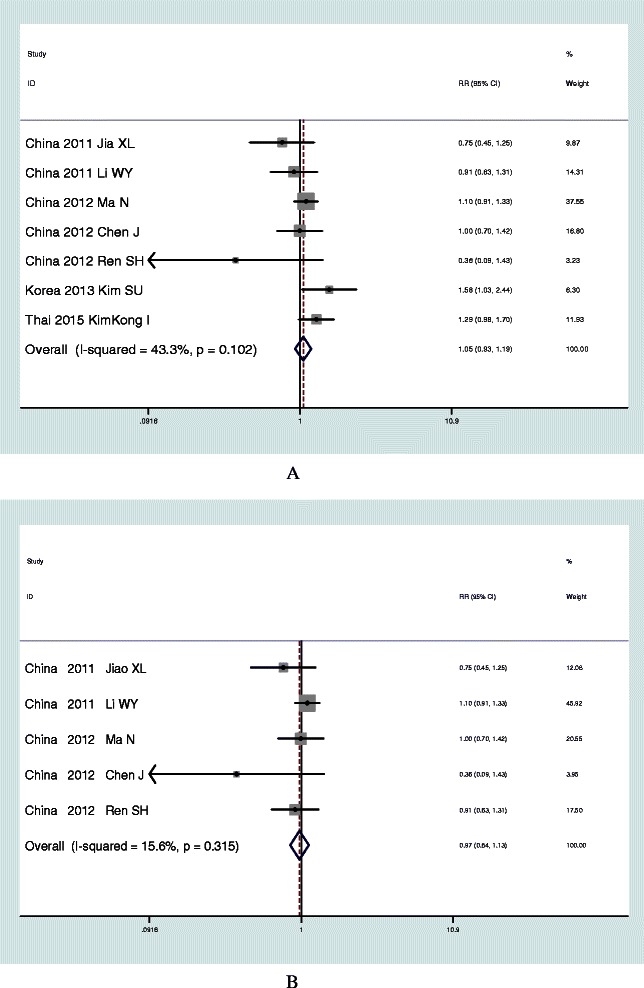
Meta-analysis results of rs8099917 (A -G) **Panel A**. Meta-analysis results of rs8099917 (A -G) in Asian population. **Panel B**. Meta-analysis results of rs8099917 (A-G) in Chinese population.

In the meta-analysis of rs8099917, the pooled sample included 3,613 subjects with HBV infection and 1,409 control subjects from 7 studies in Asian population. There was no heterogeneity among studies by Cochran Q test (P > 0.1, I^2^ = 43.3%) and a fixed effect model was performed under an additive model. The pooled OR of risk allele T was 1.05 (95% CI, 0.93-1.19; P = 0.44). No publication bias was found in the meta-analysis (corrected ɀ =0.9 and corrected p = 0.37) (Figure 3A, Table 3).

### Results of meta-analysis of rs8099917 in Chinese population

In the meta-analysis of rs8099917, the pooled sample included 3,048 subjects with HBV infection and 1,145 control subject from 5 studies in Chinese population. There was no heterogeneity among studies by Cochran Q test (P > 0.1, I^2^ = 15.6%) and a fixed effect model was performed under an additive model. The pooled OR of risk allele T was 0.97 (95% CI, 0.84-1.23; P = 0.726). No publication bias was found in the meta-analysis (corrected ɀ = 1.71 and corrected p = 0.09) (Figure 3B, Table 3).

## Discussion

IL-28B belongs to the type III IFN family, and encodes a cytokine related to type I interferons and the IL-10 family. IL-28B, IL28A and IL29 are three closely related cytokine genes located on 19q13. Expression of the three genes can be induced by viral infection. Several GWAS studies on the SNP loci affecting antiviral therapy of HCV, and multiple SNPs on the IL28B gene were associated with sustained virological response (SVR). The link was strongest among rsl2979860 3 kb upstream of IL28B gene and rs8099917 8 kb upstream of the IL28B gene. Rsl2979860 is significantly associated with the response to Peg-IFN and ribavirin for patients with chronic HCV infection. In a study to investigate the association between rs12979860 and the end-of-treatment response (ETR) or SVR in the Chinese Han population, genotype CC was the main genotype (87.64%). The patients with genotype CC had higher rates of ETR and SVR than the patients with CT or TT genotype [6]. There are differences in response to treatment between patients of European ancestry and African-Americans. In patients of European ancestry, the CC genotype is associated with a twofold greater rate of SVR than the TT genotype, threefold in African- American and twofold in Hispanic population [7]. CC genotype of rsl2979860 strongly enhances resolution of HCV infection among individuals of both European and African ancestry [8]. The studies above suggest that the effect of rsl2979860 differs in races of diverse genetic background.

In Chinese patients with dual chronic infection with hepatitis B and C viruses, rs8099917 TG genotype could increase the risk of null virological response (NVR) (OR =2.37, P =0.017), and the GG genotype had a further increased risk of NVR (OR = 4.23, P = 0.027). The rs12979860 allele was associated with treatment failure (CT/TT vs. CC: OR =2.04, P =0.037). IL28B rs8099917 G variant leads to higher risk of NVR (P = 0.009) in HCV genotype 1 [8]. In GWAS to NVR in the treatment of patients with HCV genotype 1 of Japanese population, rs8099917 was strongly associated with NVR and SVR [9].

The relationship between IL28B and HCV is widely studied, but the association study between IL28B and HBV infection is much less and the results were inconsistent in previous studies. In two studies of rs12979680 on infection of hepatitis B virus in Chinese people, there was no association between rs12979680 and HBV infection [10,11]. In another Chinese study, both C allele and CC genotype were protective, which indicated that the rs12979860 TC polymorphism is associated with the carcinogenic process of chronic hepatitis B and HBV-related HCC [12]. In a meta-analysis of rs12979680, no significant correlation with HBV infection outcomes was found, and the pooled OR was 0.42 (95% CI, 0.11-1.51; P = 0.184, I^2^ = 75.9%) in Asian population [13]. But in our study, T allele of rs12979680 can increase the risk of HBV infection in Chinese population but not Asian population under an additive genetic model, the pooled OR (95% CIs) were 1.62 (1.04–2.51). Compared with the previous meta-analysis of rs12979680 (the included three papers was updated in July 2013) [14], our study contains more papers and more sample size. In the seven papers of our meta-analysis, there are five studies on Chinese population, one on Korea and one on Thai population, the frequency of T allele is 6.7 ~ 12.8% in Chinese population which is higher compared with 5.5% in Korea population and 6.4% in Thai population. The difference in the frequency of T allele in China, Korea and Thai may explain the inconsistent result in the meta-analysis on the Chinese population and the Asian population. However, the papers included in the meta-analysis are not enough and the result deserves more research to testify.

In our meta-analysis of rs8099917, there is no association between rs8099917 and susceptibility of HBV in both Chinese population and the Asian population. Jiao et al. demonstrate that rs8099917 is not associated with the HBV infection [11], which is consistent with our result. In another research, rs8099917 is strongly associated with glutamic-pyruvic transaminase (ALT) level and HBV viral load [10] IL28B rs8099917 AA genotype (AA vs AC + CC: odds ratio (OR) = 0.63) was associated with a decreased risk of HCC [13]. In a Korea study, rs8099917 is significantly associated with the outcomes of HBV infection [15]. The result deserves more research to testify.

### Study limitations

There are several limitations that should be stated. First, the frequency of the allele is not classified by the hepatitis B virus genotypes which are different in different countries. Second, only seven papers were included in the meta-analysis, and so the association between IL28B (rs12979680 and rs8099917) and infection susceptibility of HBV in this meta-analysis deserves further replication.

## Conclusions

In summary, our study demonstrated that T allele of rs12979680 can increase the risk of HBV infection in Chinese population but not Asian population under an additive genetic model. There is no association between rs8099917 HBV infection in Chinese population and Asian population.

## Abbreviations

HBV: Hepatitis B virus
WHO: World Health Organization
IL28B: Interleukin 28B
GWAS: Genome-wide association studies
SNPs: Single nucleotide polymorphisms
HWE: Hardy-Weinberg equilibrium
OR: Odds ratios
CNKI: China National Knowledge infrastructure
SVR: Sustained virological response
ETR: End of treatment response
NVR: Null virological response
ALT: Glutamic-pyruvic transaminase
